# Retinal Thickening and Photoreceptor Loss in HIV Eyes without Retinitis

**DOI:** 10.1371/journal.pone.0132996

**Published:** 2015-08-05

**Authors:** Cheryl A. Arcinue, Dirk-Uwe Bartsch, Sharif Y. El-Emam, Feiyan Ma, Aubrey Doede, Lucie Sharpsten, Maria Laura Gomez, William R. Freeman

**Affiliations:** 1 Jacobs Retina Center at the Shiley Eye Institute, University of California San Diego (UCSD), La Jolla, California, United States of America; 2 Ophthalmology, Tanta University, Tanta, Egypt; The Forsyth Institute, UNITED STATES

## Abstract

**Purpose:**

To determine the presence of structural changes in HIV retinae (i.e., photoreceptor density and retinal thickness in the macula) compared with age-matched HIV-negative controls.

**Methods:**

Cohort of patients with known HIV under CART (combination Antiretroviral Therapy) treatment were examined with a flood-illuminated retinal AO camera to assess the cone photoreceptor mosaic and spectral-domain optical coherence tomography (SD-OCT) to assess retinal layers and retinal thickness.

**Results:**

Twenty-four eyes of 12 patients (n = 6 HIV-positive and 6 HIV-negative) were imaged with the adaptive optics camera. In each of the regions of interest studied (nasal, temporal, superior, inferior), the HIV group had significantly less mean cone photoreceptor density compared with age-matched controls (difference range, 4,308–6,872 cones/mm^2^). A different subset of forty eyes of 20 patients (n = 10 HIV-positive and 10 HIV-negative) was included in the retinal thickness measurements and retinal layer segmentation with the SD-OCT. We observed significant thickening in HIV positive eyes in the total retinal thickness at the foveal center, and in each of the three horizontal B-scans (through the macular center, superior, and inferior to the fovea). We also noted that the inner retina (combined thickness from ILM through RNFL to GCL layer) was also significantly thickened in all the different locations scanned compared with HIV-negative controls.

**Conclusion:**

Our present study shows that the cone photoreceptor density is significantly reduced in HIV retinae compared with age-matched controls. HIV retinae also have increased macular retinal thickness that may be caused by inner retinal edema secondary to retinovascular disease in HIV. The interaction of photoreceptors with the aging RPE, as well as possible low-grade ocular inflammation causing diffuse inner retinal edema, may be the key to the progressive vision changes in HIV-positive patients without overt retinitis.

## Introduction

Visual function abnormalities are common in human immunodeficiency virus (HIV) infected patients without retinitis even after improvement in immune status with anti-retroviral therapy.[1,2] These abnormalities include reduced contrast sensitivity [3], altered color vision, peripheral visual field loss, and electrophysiological changes.[4–9] These abnormalities are thought to be due to damage to the retinal nerve fiber layer (RNFL) and secondary peripapillary changes[10], most probably due to micro-infarctions and microangiopathy.[11–13] Our group was the first to describe the RNFL in patients with HIV.[11] We found that patients with HIV had significantly thinner peripapillary RNFL in the temporal, superior, and inferior quadrants without any evidence of glaucomatous changes. Other groups have confirmed our findings over the years.[3,14,15]

We have also shown that there is permanent structural damage in such patients using *in vivo* measurements of retinal tissue by OCT and other imaging modalities. Our most recent studies of autopsy tissue have used microarray based analysis to show that there is an induction of rhodopsin and other transcripts involved in visual transduction, as well as structural components of the rod photoreceptors.[16]

Adaptive optics (AO) has recently achieved success in a range of applications in ophthalmology. After its initial development for astronomy, it has been integrated into flood illumination full-field retinal cameras, confocal scanning laser ophthalmoscopes (SLO), and optical coherence tomography (OCT) instruments for high-resolution reflectance imaging.[17–26] AO-corrected imaging of the posterior pole allows the imaging instrument to obtain high-resolution images near the levels of resolution that are otherwise common in microscope-based biological studies. AO imaging is now available with a flood-illumination camera intended for research use only.[27,28]

This study aims to determine if there are changes in retinal structure that may be associated with HIV disease, particularly in patients with a large HIV disease burden as assessed by a history of a CD4 T cell count below 100 cells/mm^3^, a threshold previously used by our group [9]^,^ [11]^,^ [29]^,^ [30]^,^ [31]. We wished to determine if there were differences in the mean density of the cone photoreceptor mosaic in the macula of HIV-positive patients with low CD4 nadir T cell counts (without ocular opportunistic infections) compared with age-matched normal controls using adaptive optics (AO) imaging. The second aim of this study is to compare the macular retinal thickness and retinal layer segmentation between HIV-positive and normal controls using spectral-domain optical coherence tomography (SD-OCT).

## Materials and Methods

Patients with known HIV under CART (combination Antiretroviral Therapy) treatment were examined with a flood-illuminated retinal AO camera (rtx-1, Imagine Eyes, Orsay, France) to assess the cone photoreceptor mosaic and spectral-domain optical coherence tomography (SD-OCT) (Spectralis HRA+OCT, Heidelberg Engineering, Carlsbad, CA) to assess retinal layers and retinal thickness at the Jacobs Retina Center, University of California San Diego (UCSD) Shiley Eye Center. Written informed consent was obtained for each patient prior to the AO and SD-OCT imaging procedures. Institutional Review Board (IRB) approval was acquired for the review and analysis of patient data. The study adhered to the tenets of the Declaration of Helsinki. The UCSD Human Research Protection Program is the approving IRB and the IRB number is 130568.

### Study Population

HIV-positive patients under CART treatment were imaged with the AO camera and SD-OCT. Inclusion criteria included the following: (1) history of low CD4 nadirs, (2) ability to fixate well, (3) no history of CMV retinitis. Age-matched controls were selected and imaged using the same protocol.

### Scanning Protocol Using the rtx1 Adaptive Optics Retinal Camera

The rtx1 is an adaptive optical fundus camera using flashed, non-coherent flood illumination with 2 light sources. A low-coherence superluminescent diode centered at 750 nm used for measuring and correcting optical aberrations and controlling the focus at the retinal layers, and a light-emitting diode with a wavelength centered at 850 nm for uniform illumination of the retinal area imaged.[32] The instrument is claimed to have a pixel resolution of 1.6 microns and has the ability to resolve 250 line pairs per millimeter. Lombardo and colleagues reported that the very center of the fovea (<0.2 mm eccentricity) cannot be accurately resolved with the rtx1 [33]. The instrument uses a yellow fixation cross to help the subject fixate. The location of the center of the fixation cross is stored by the instrument, which permits the subject to fixate on the same stimulus location.

**Fig 1:**
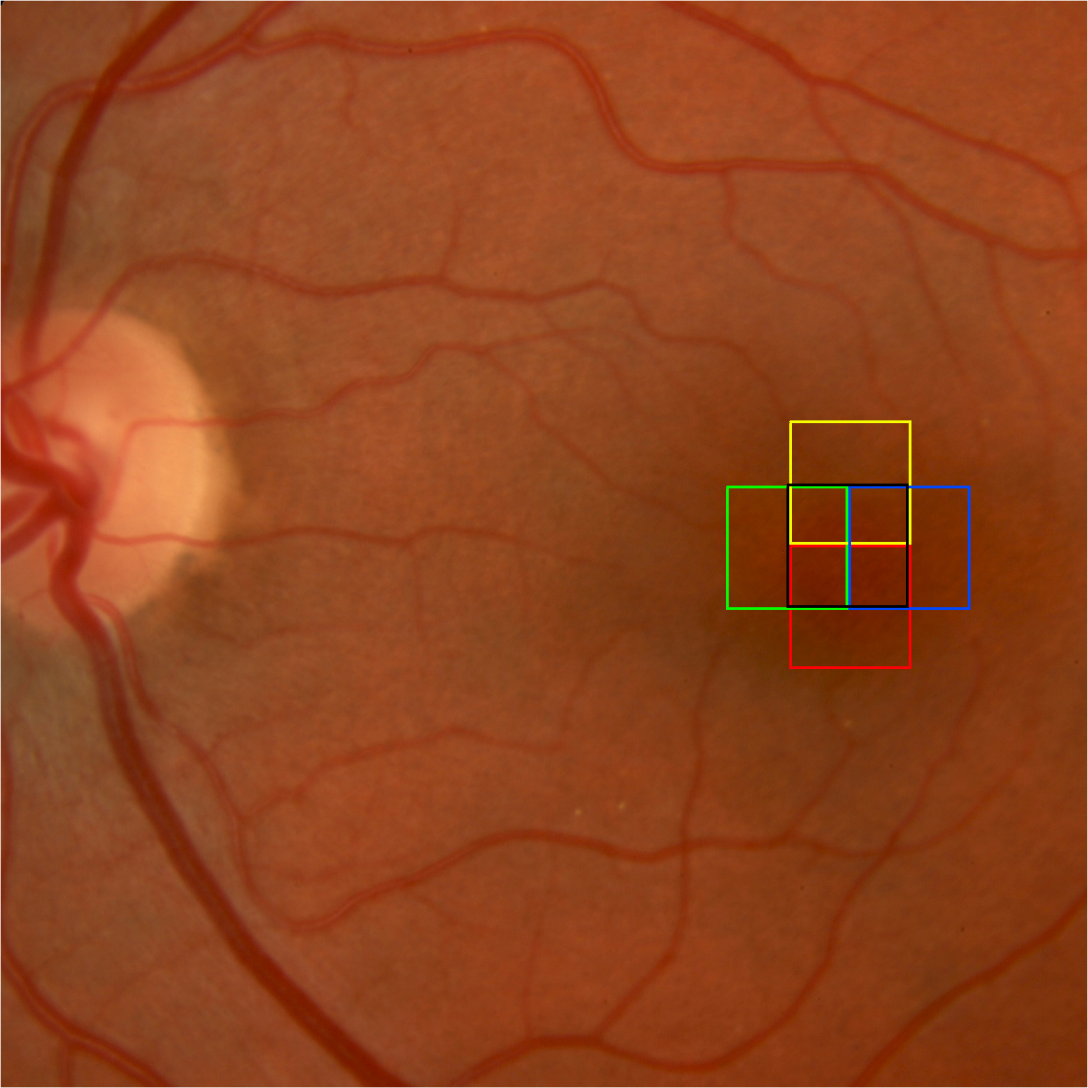
Color fundus photo with boxed areas showing the different locations of the fovea and perifoveal areas that were scanned using the adaptive optics camera with the patient fixating between 0 degrees and 2 degrees of retinal eccentricity from the foveal center along the horizontal and vertical meridians.

All the study eyes were dilated for the AO exam. Twelve eyes of 6 patients in each group were examined. Each image was obtained from an average of 40 frames of a 4° x 4° retinal area over an acquisition time of 4 seconds. The patient was asked to keep good fixation on the intersection of the crossed lines. Multiple images of different locations of the fovea and perifoveal areas were scanned with the patient fixating between 0 degrees and 2 degrees of retinal eccentricity from the foveal center along the horizontal and vertical meridians (Fig 1), as follows: 2° nasal/0° vertical, 2° temporal/0° vertical, 2° superior/0° horizontal, 2° inferior/0° horizontal. The AO camera was carefully focused through the depth of the retina to detect the highest reflectivity consistent with the cone inner segments.

### Postprocessing of the Adaptive Optics Images

**Fig 2:**
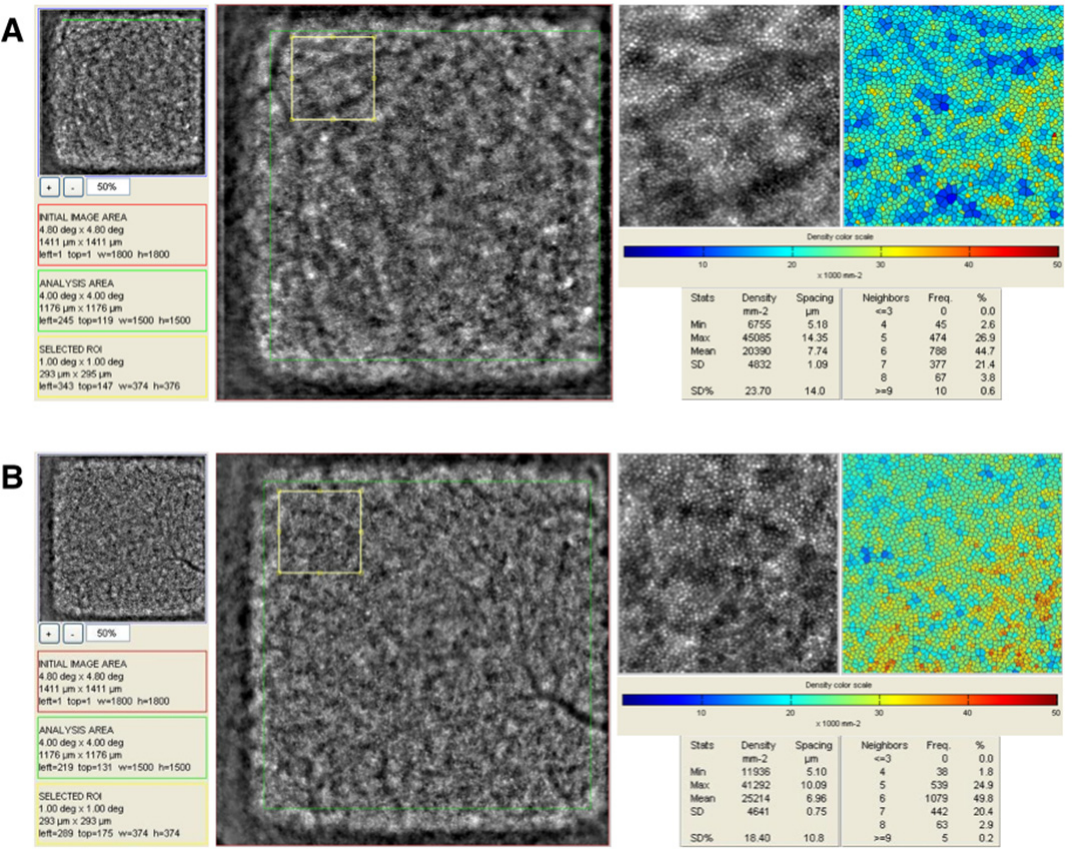
Postprocessing of Adaptive Optics Images. A. HIV-positive, and B. Age-matched control, with scan centered on the fovea (fixation at 0 deg). Selected region of interest (ROI) (small yellow box). ROI magnified with corresponding color map of the cone density. Photoreceptor density counts shown.

Each series of 40 images acquired by the AO camera was processed using original software programs, provided by the manufacturer (CK v0.1 and AOdetect v0.1, Imagine Eyes). These images were registered and averaged to produce a final image with improved signal-to-noise ratio. The histogram of the resulting averaged image was stretched over a 16-bit range of gray levels for display purposes. The positions of photoreceptor inner segments were computed by automatically detecting the central coordinates of small circular spots whose brightness differed from the surrounding background level. The spatial distribution of these point coordinates was finally analyzed in terms of local cell numerical density (cells per square millimeter of retinal surface)[32]. For each patient, cone packing density analysis was performed on AO images within a range of 0.5° x 0.5° windows to 1° x 1° windows in the 4 regions of interest (ROI) (nasal, temporal, superior, inferior). Smaller ROI were chosen to avoid areas where the machine cannot count the cone photoreceptors, such as blood vessels. Each ROI chosen for each location (nasal, temporal, superior, inferior) was matched exactly to the same area in the eyes of the matched controls (Fig 2).

### Segmentation of Spectral-Domain Optical Coherence Tomography (SD-OCT) Scans

**Fig 3:**
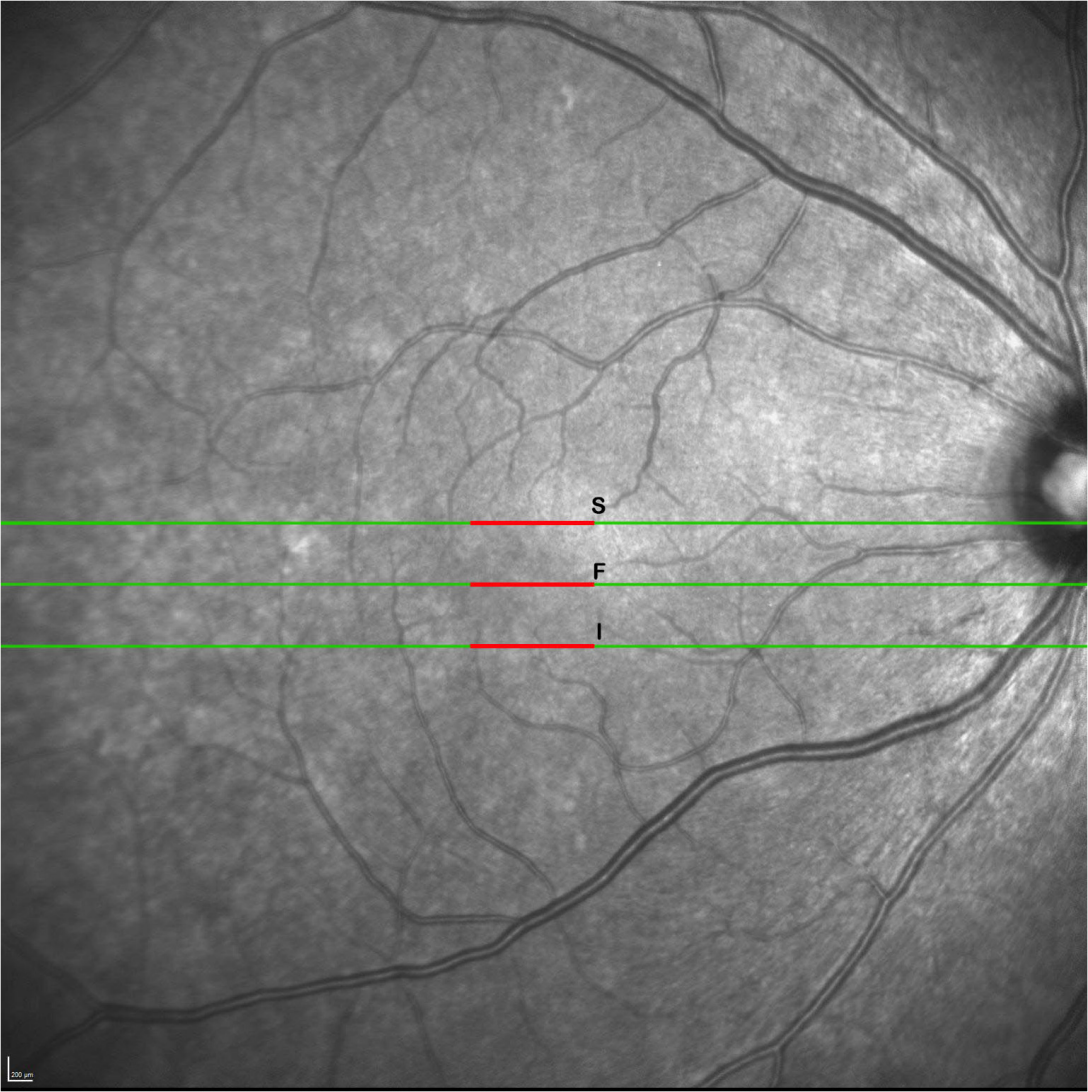
Analysis of three B-scans within the raster scan set. The first was centered on the fovea (F), the second was 10 scans superior to the fovea (S), and the third was 10 scans inferior to the fovea (I). For each scan, thickness was measured at the center and over a length of 1,000 microns equally split over the center (red lines).

**Fig 4:**
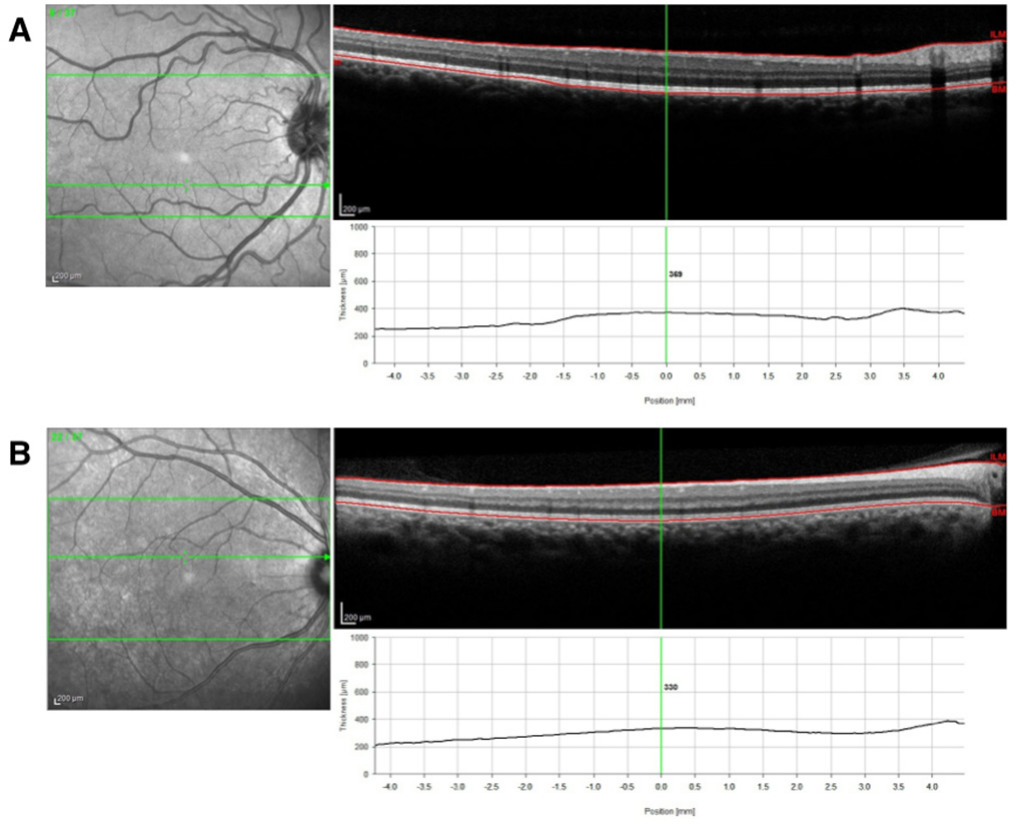
SD-OCT scans of A. HIV-positive, and B. HIV-negative control, showing total retinal thickness measurements. Top red line corresponds to the internal limiting membrane (ILM) and bottom red line corresponds to the Bruch’s membrane (BM).

For a separate subset of patients (n = 10 HIV+ and 10 HIV-), we acquired raster scan pattern high-resolution SD-OCT scans (30° horizontal x 15° vertical, 1,536 A-scans/B-scan, sampling interval 127 μm) using the Heidelberg Spectralis. The OCT images were reviewed using original software from Spectralis. We analyzed three B-scans within the raster scan set (Fig 3). The first was centered on the fovea, the second was 10 scans superior to the fovea (1,270 microns), and the third was 10 scans inferior to the fovea (1,270 microns). For each scan, we measured the thickness at the center of the scan and the average thickness over a length of 1,000 microns equally split over the center of the scan. We used prototype segmentation software to analyze the individual layers of the retina and manually corrected artifacts within the segmentation. In this study, we calculated several measurements based on the segmentation. The first measurement was total retinal thickness, defined as the distance between the internal limiting membrane (ILM) and the Bruch’s membrane (BM). We defined the inner retina as the distance between the internal limiting membrane and the posterior edge of the ganglion cell layer (GCL). The outer retina was defined as the distance between the external limiting membrane (ELM) and the Bruch’s membrane (BM), including the array of photoreceptor inner segments (ISOS) [34]. The different layer interfaces that were segmented by the software were in order from anterior to posterior: ILM, retinal nerve fiber layer (RNFL), GCL, inner plexiform layer (IPL), inner nuclear layer (INL), outer plexiform layer (OPL), outer nuclear layer (ONL), ISOS and BM (Fig 4). We calculated a total of 8 layer thicknesses and 3 combination layer thicknesses (total, outer, inner) and the mean retinal thickness along a 1,000-micron lateral distance for the superior and inferior scan line and for the foveal center at the scan centered on the fovea. Thus, for each of the three scan lines, we calculated 12 retinal thickness measurements.

### Statistical Analysis

Descriptive statistics included the mean and standard deviation for continuous variables. The Wilcoxon signed-rank test was used to evaluate differences in the photoreceptor densities in the 5 regions of interest (fovea, nasal, temporal, superior, inferior) and the retinal thickness measurements between HIV-positive eyes compared with age-matched controls. All statistical analyses were performed using SAS software version 9.3 (SAS Institute, Cary, North Carolina, USA). The α level (type I error) was set at 0.05.

## Results

Twenty-four eyes of 12 patients (n = 6 HIV-positive and 6 HIV-negative) were imaged with the adaptive optics camera (S1 Table). One hundred percent (n = 6) of the HIV-positive patients were males, and 50% (n = 3) were males in the control group. The mean age of the HIV group was 52.3 ± 5.4 years (range, 45–61) while the mean age of the matched control group was 45.3 ± 5.6 years (range, 36–52). The difference in the mean age between the 2 groups was statistically significant (p = 0.031) (Table 1).

**Table 1:** Baseline demographic characteristics.

	HIV-positive group	HIV-negative group	P-value
Photoreceptor Density (Adaptive Optics)			
Number of patients (eyes)	6 (12)	6 (12)	
Age in years, mean ± SD	52.3 ± 5.4	45.3 ± 5.6	**0.031**
Sex (males)	6	3	
Retinal Thickness (SD-OCT)			
Number of patients (eyes)	10 (20)	10 (20)	
Age in years, mean ± SD	52.1 ± 12.2	55.8 ± 14.4	0.370
Sex (males)	9	6	
Mean refractive error, mean ±SD	+1.01 ± 1.51	+0.90 ± 1.48	0.834

Table 2 illustrates the mean cone photoreceptor density in the 5 different regions of interest in both groups. The mean foveal cone density in the HIV group was 16967 ± 2810 cones/mm^2^ compared with the control group of 22744 ± 2331 cones/mm^2^. There is a difference of 5777 cones/mm^2^ (p = 0.002). Inferior to the fovea, the mean cone density was 16004 ± 3056 cones/mm^2^ in the HIV group compared with 22876 ± 2731 cones/mm^2^ in the control group (p = 0.016). Superior to the fovea, the HIV group had a mean cone density of 14780 ± 2847 cones/mm^2^ while the control had a mean density of 20692 ± 2112 cones/mm^2^ (p = 0.016). Similar differences were also noted in the nasal (HIV group 15932 ± 3935 vs. control group 22218 ± 3556) and temporal (HIV group 16752 ± 3626 vs. control group 21060 ± 2527) regions (p = 0.016). In each of the regions of interest studied, the HIV group had significantly less mean cone photoreceptor density compared with age-matched controls (difference range, 4308–6872 cones/mm^2^).

**Table 2:** Mean Cone Photoreceptor Density in the Macula of HIV-Positive Individuals vs. Matched Controls.

Region of Interest (ROI)	Mean Cone Density ± Standard Deviation		Difference (cones/mm^2^) (% difference)	P-value
	HIV-positive (Low CD4 counts) (cones/mm^2^)	HIV-negative (Matched controls) (cones/mm^2^)		
Inferior	16004 ± 3056	22876 ± 2731	-6872 (-30%)	**0.016**
Superior	14780 ± 2847	20692 ± 2112	-5912 (-29%)	**0.016**
Nasal	15932 ± 3935	22218 + 3556	-6286 (-28%)	**0.016**
Temporal	16752 ± 3626	21060 ± 2527	-4308 (-20%)	**0.016**

Forty eyes of 20 patients (n = 10 HIV-positive and 10 HIV-negative) were included in the retinal thickness measurements and retinal layer segmentation with the SD-OCT (S2 Table). Our HIV-positive group had an average age of 52.1 ± 12.2 years, and the HIV-negative group had an average age of 55.8 ± 14.4 years. The age difference was not significant (p = 0.370). The group of HIV-positive patients consisted of 9 males and one female while the HIV-negative patients consisted of 6 males and 4 females. Average refractive error was +1.01 ± 1.51 (range, -2.24 to +2.85) for the HIV-positive group, and +0.90 ± 1.48 (range, -3.35 to +2.76) for the HIV-negative group (p = 0.834) (Table 1). Our analysis of the SD-OCT data revealed a significant difference in the total retinal thickness in several locations. The HIV positive group had a thicker average retinal thickness at the foveal center of 232.6 ± 23.4 microns, compared with the matched HIV negative control group that had an average thickness of 213.1 ± 14.5 microns (p = 0.001) (Table 3). Similar findings of significantly increased total retinal thickness in the HIV-positive eyes were also noted across the macular center of a 1000-micron long scan (+24.4 microns, p<0.0001), superior (+12.1 microns, p = 0.033), and inferior to the fovea (+15.9 microns, p<0.0001), compared with the HIV-negative controls (Tables 4–6). When we analyzed the different retinal layers and the group of inner retinal layers and outer retinal layers, we found a significantly thicker inner retina in the HIV-positive eyes at the scans going 1000 microns across the macular center (+8.6 microns, p = 0.012), superior (+4.3 microns, p = 0.043), and inferior to the fovea (+6.6 microns, p = 0.039), compared with HIV-negative controls (Tables 4–6).

**Table 3:** Retinal Thickness Measurements of HIV-Positive Individuals vs. Matched Controls (Point Measurement at Foveal Center).

Retinal layers	Mean Retinal Thickness ± Standard Deviation		Difference (microns)	P-value
	HIV-positive (Low CD4 counts) (microns)	HIV-negative (Matched controls) (microns)		
Total retina at foveal center	232.6 ± 23.4	213.1 ± 14.5	+19.5	**0.001**

**Table 4:** Retinal Thickness Measurements of HIV-Positive Individuals vs. Matched Controls in Horizontal OCT B-scan Through the Macular Center.

Retinal layers	Mean Retinal Thickness ± Standard Deviation		Difference (microns)	P-value
	HIV-positive (Low CD4 counts) (microns)	HIV-negative (Matched controls) (microns)		
Total retina	268.5 ± 20.3	244.1 ± 11.2	+24.4	**<0.0001**
Outer retina	86.0 ± 7.4	82.4 ± 13.0	+3.6	0.404
Inner retina	40.6 ± 10.9	32.0 ± 12.8	+8.6	**0.012**
RNFL	32.5 ± 10.3	25.2 ± 34.1	+7.3	0.459
GCL	19.0 ± 12.7	9.3 ± 4.5	+9.7	**0.003**
IPL	3.2 ± 26.9	7.0 ± 33.9	-3.8	0.821
INL	41.0 ± 41.3	28.0 ± 39.8	+13.0	0.165
OPL	19.6 ± 5.8	14.8 ± 3.9	+4.8	**0.005**
ONL	87.6 ± 29.0	87.7 ± 32.2	-0.01	0.961
ISOS	32.1 ± 5.5	30.8 ± 5.3	+1.3	0.307
BM	47.7 ± 16.3	48.8 ± 16.5	-1.1	0.746

RNFL–retinal nerve fiber layer; GCL–ganglion cell layer; IPL–inner plexiform layer; INL–inner nuclear layer; OPL–outer plexiform layer; ONL–outer nuclear layer; ISOS–array of photoreceptor inner segments; BM–Bruch’s membrane; outer retina: ILM+RNFL+GCL; inner retina: ELM+ISOS+BM

**Table 5:** Retinal Thickness Measurements of HIV-Positive Individuals vs. Matched Controls in Horizontal OCT B-scan at Location Superior to the Fovea.

Retinal layers	Mean Retinal Thickness ± Standard Deviation		Difference (microns)	P-value
	HIV-positive (Low CD4 counts) (microns)	HIV-negative (Matched controls) (microns)		
Total retina	341.1 ± 34.1	329 ± 15.5	+12.1	**0.033**
Outer retina	74.9 ± 9.5	70.8 ± 7.8	+4.1	0.234
Inner retina	131.4 ± 15.1	127.1 ± 9.3	+4.3	**0.043**
RNFL	36.5 ± 5.5	36.0 ± 5.2	+0.5	0.942
GCL	48.3 ± 12.1	47.4 ± 10.3	+0.9	0.819
IPL	45.4 ± 9.9	43.1 ± 11.7	+2.3	0.441
INL	42.3 ± 7.1	42.8 ± 4.5	-0.5	0.715
OPL	28.9 ± 14.4	29.1 ± 19.1	-0.2	0.805
ONL	70.1 ± 13.0	63.6 ± 11.2	+6.5	0.087
ISOS	29.1 ± 4.7	27.6 ± 3.6	+1.5	0.281
BM	44.6 ± 11.1	43.7 ± 8.1	+0.9	0.763

RNFL–retinal nerve fiber layer; GCL–ganglion cell layer; IPL–inner plexiform layer; INL–inner nuclear layer; OPL–outer plexiform layer; ONL–outer nuclear layer; ISOS–array of photoreceptor inner segments; BM–Bruch’s membrane; outer retina: ILM+RNFL+GCL; inner retina: ELM+ISOS+BM

**Table 6:** Retinal Thickness Measurements of HIV-Positive Individuals vs. Matched Controls in Horizontal OCT B-scan at Location Inferior to the Fovea.

Retinal layers	Mean Retinal Thickness ± Standard Deviation		Difference (microns)	P-value
	HIV-positive (Low CD4 counts) (microns)	HIV-negative (Matched controls) (microns)		
Total retina	338.6 ± 24.7	322.7 ± 16.4	+15.9	**<0.0001**
Outer retina	73.6 ± 4.9	69.2 ± 8.0	+4.4	0.116
Inner retina	131.1 ± 15.7	124.5 ± 10.0	+6.6	**0.039**
RNFL	35.3 ± 6.0	36.4 ± 6.1	-1.1	0.833
GCL	48.9 ± 9.0	59.3 ± 38.9	-10.4	0.589
IPL	45.5 ± 9.1	39.3 ± 12.1	+6.2	0.051
INL	43.1 ± 4.6	44.0 ± 15.2	-0.9	0.264
OPL	24.8 ± 4.3	23.3 ± 4.2	+1.5	0.233
ONL	65.8 ± 8.9	63.0 ± 14.3	+2.8	0.667
ISOS	27.8 ± 3.5	28.8 ± 4.6	-1.0	0.586
BM	46.5 ± 6.6	39.3 ± 11.1	+7.2	0.089

RNFL–retinal nerve fiber layer; GCL–ganglion cell layer; IPL–inner plexiform layer; INL–inner nuclear layer; OPL–outer plexiform layer; ONL–outer nuclear layer; ISOS–array of photoreceptor inner segments; BM–Bruch’s membrane; outer retina: ILM+RNFL+GCL; inner retina: ELM+ISOS+BM

## Discussion

One of the aims of this study was to detect any differences in the mean density of the cone photoreceptor mosaic in the macula of HIV-positive patients under highly active anti-retroviral therapy (CART) compared with age-matched normal controls using adaptive optics (AO) imaging. In each of the regions of interest studied (fovea, nasal, temporal, superior, inferior), the HIV group had significantly less mean cone photoreceptor density compared with age-matched controls (difference range, 4308–6872 cones/mm^2^). Our measurements were taken between 0° and 4° eccentricity. Lombardo and co-workers studied the resolution limit of the ImagineEyes rtx1 and found that the center of the fovea (<0.2 mm eccentricity) was beyond the detection limit of the instrument [33]. Therefore, we limited our measurements to the region outside of the central 0.2 mm. Burns and coworkers studied the normal distribution of cone packing density and found that the density was predominantly dependent on eccentricity and secondary on quadrant.[35] They reported packing densities of between 22,000 and 29,100 cones/mm^2^ at 2.4° and 15,200 and 20,900 cones/mm^2^ at 3.6° eccentricity. Thus, our results are within the range provided by Burns and coworkers.

Because of the small sample size of our subgroup analysis of photoreceptor density, there was not enough power to correct for age, and we tried to age-match our HIV patients as closely as we can to normal controls. Although there is a 7-year difference in the average age of our two groups that is statistically significant, the difference in the photoreceptor density between the 2 groups ranges from 20–30%. Park et al[36] found that the effect by aging is only slight. They have shown only a 3% change at 0.5 mm eccentricity between the < 20-year-old group and the > 50-year-old group. Our present finding of a 20–30% difference in photoreceptor density between the HIV-positive group and normal control group is definitely significant, and not resulting from age difference alone. Burns and coworkers studied the age-dependency in two groups of subjects. The younger group was between 22 and 35 years of age, while the older group was between 50 and 65 years of age. They observed a significant difference between both groups only at eccentricities of less than 0.45 mm (equivalent to 1.5°). Furthermore, the age difference between their groups was much larger than in our case. Additionally, our measurements were outside the foveal center where Burns did not find an age-dependent variability.

Changes seen in the outer retina, specifically in the retinal pigment epithelium (RPE) and photoreceptor outer segments contribute to vision changes in non-infectious HIV retinopathy. We have previously reported that in HIV-positive retinae, there is an induction of rhodopsin and other transcripts (including *PDE6A*, *PDE6B*, *PDE6G*, *CNGA1*, *CNGB1*, *CRX*, *NRL*) involved in visual transduction, as well as structural components of the rod photoreceptors (*ABCA4* and *ROM1*). This is consistent with an increased rate of renewal of rod outer segments induced via increased phagocytosis by HIV-infected RPE previously reported in culture. Cone-specific transcripts (*OPN1SW*, *OPN1LW*, *PDE6C*, *PDE6H* and *GRK7*) are uniformly down-regulated in HIV positive retinae, likely due to a partial loss of cone photoreceptors.[16] This correlates with our present finding of decreased cone photoreceptor density in HIV positive retinae compared with age-matched controls.

The second aim of this study was to compare the thickness of the different retinal layers in the macula using manual segmentation of the SD-OCT scans between HIV-positive and normal controls. We observed a significant thickening in HIV positive subjects in the total retinal thickness at the foveal center and each of our three horizontal B-scans. We also noted that the inner retina (combined thickness from ILM through RNFL to GCL layer) was also significantly increased. We also found that there was only a significant difference in the thickness of the GCL and OPL retinal layers along the B-scan through the macular center. No significant difference was found in other retinal layers in the B-scan through the macular center or any of the individual retinal layers in the B-scans superior and inferior to the macula. Most of them showed a trend towards thickening in our HIV positive subjects.

In the Singapore Chinese Eye Study[37] analyzing macular thickness of 490 patients aged between 40–80 years using SD-OCT (Cirrus OCT), they found a small decrease in macular thickness with age of 0.38 microns per year of age. Therefore, between the ages of 40 and 80 years, the macular thickness changes by only 15 microns. In our subpopulation comparing retinal thickness between HIV positive and negative retinae, we had a non-significant age difference of 3 years between the 2 groups, which amounts to approximately 1-micron change in retinal thickness due to the slight age difference between the groups. Our HIV-negative group had decreased retinal thickness and a slightly older average age compared with the HIV-positive group. It is known that age is associated with thinner retinal thickness, but this association only explains about 1.4 microns of change. Our present finding of +19.5 microns change in average retinal thickness at the foveal center between the 2 groups is certainly significant and not due to age difference alone. Aside from age, Gupta et al[37] also noted that thinner overall average macular thickness was associated with female sex (4.46 micron thinner compared to males) and longer axial length (AL) (2.34 micron decrease per each mm increase in AL). If we add 4.46 micron to each measurement taken in a female, the average retinal thickness in our HIV negative group increases by only 1.6 micron. Thus, the slight gender mismatch cannot explain the observed difference of +19.5 microns. Furthermore, in the present study, the refractive error between the two groups was not statistically different (-0.108, p = 0.834) and thus does not contribute to the difference noted. We recently reported on the degenerative retinal process in patients with HIV-associated non-infectious retinopathy[16]. We found significant outer retinal dysfunction, molecular changes in the outer retina and a cascade of up-regulation of rod outer segment mRNA markers. We believe that this dysfunction could explain the diffuse retinal thickening. Alternative explanations such as age, sex and refractive error are not sufficient to explain the large difference in retinal thickness. There are several retinal pathologies that have demonstrated to cause retinal thickening as measured by OCT. These include central retinal artery occlusion [38], infectious retinitis [39], X-linked retinoschisis [40], cat-scratch disease neuroretinitis [41], and diabetes with macular edema [42] while other disease are associated with retinal thinning such as diabetes without macular edema [42]. Iester and colleagues suggested that a thinning of the inner retinal layers was related to diseases that affect ciliary or retinal arterial vessels, while a reduction in the outer retinal layers may be related to pathologies related to choroidal flow diseases [43].

There is a possibility that the retinal changes observed in our study were solely or in part due to the interactions of the individual antiretroviral drugs used to treat the HIV-positive patients over a long period of time. In particular, our control population did not, understandably, receive the antiretroviral drugs. There has been speculation that certain drugs can cause retinal toxicity. These drugs with a known potential to cause anatomic damage to the retina include chloroquine, hydroxychloroquine, thioridazine, deferoxamine, tamoxifen, canthaxanthine, digoxin, interferon, rifabutin, and fomivirsen. In addition, at least one antiretroviral therapy has been shown to exhibit retinal toxicity–didanosine [44]. However, none of these drugs were used in our study population.

There is the possibility that chronic human cytomegalovirus (HCMV) infection was participating directly or indirectly in the retinal changes observed in the HIV-positive subjects [45]. While we noted that none of our subjects had retinal damage due to infectious retinitis related to CMV, many or all of our HIV-positive subjects were probably HCMV seropositive and may have harbored chronically infected patrolling monocytes in their bloodstream. However, we currently do not have the answer if CMV infected blood cells or other viruses were present in our subjects.

Our group has previously studied the RNFL thickness at the optic nerve head and found a significant reduction in peripapillary retinal nerve fiber layer thickness in HIV positive subjects compared to HIV negative controls.[11] A study by Moschos[46] using the Stratus OCT in 2011 found an increase in foveal thickness in HIV positive children compared to a group of age-matched normal controls. Although the peripapillary RNFL thickness is decreased in HIV retinae, the foveal retinal thickness is apparently thicker compared with normal controls. An alternative explanation for this is an ongoing low-grade inflammatory process in the retina of HIV-positive patients causing a mild increase in retinal thickness without any clinical manifestations. This low-grade inflammatory process causes decompensation of the RPE. In addition, the presence of sub-clinical HIV vasculopathy might possibly create a breakdown of the inner and outer blood-retinal barrier. [47–49]

## Conclusion

Our present study shows that the cone photoreceptor density is significantly reduced in HIV retinae compared with age-matched controls. HIV retinae also have increased macular retinal thickness that may be caused by inner retinal edema secondary to retinovascular disease in HIV. The interaction of photoreceptors with the aging RPE, as well as possible low-grade ocular inflammation causing diffuse inner retinal edema, may be the key to the progressive vision changes in HIV-positive patients without overt retinitis.

## Supporting Information

S1 Table Adaptive Optics Photoreceptor Density. Group A is the HIV-positive group, Group B is the HIV-negative group. Columns ending in A are for group A. Columns ending in B are for group B. IDA and IDB have the ID numbers for groups A and B, respectively. EyeA and EyeB show which eye was examined for groups A and B, respectively. NasalA and NasalB show the cone photoreceptor count for nasal quadrant for groups A and B, respectively. TemporalA and TemporalB show the cone photoreceptor count for temporal quadrant for groups A and B, respectively. SuperiorA and SuperiorB show the cone photoreceptor count for superior quadrant for groups A and B, respectively. InferiorA and InferiorB show the cone photoreceptor count for inferior quadrant for groups A and B, respectively.
(XLSX)

S2 Table Retinal Thickness Values for Segmented Layers. Group A is the HIV-positive group, Group B is the HIV-negative group. Columns starting with A are for group A. Columns starting with B are for group B. A-ID and B-ID have the ID numbers for groups A and B, respectively. A-Eye and B-Eye show which eye was examined for groups A and B, respectively. The next column shows the raw data for retinal thickness at fovea center (Fovea center). The next columns show the data along the line through the macular center for total retinal thickness, outer retina, inner retina, RNFL, GCL, IPL, INL, OPL, ONL, ISOS layer and BM layer. The subsequent columns show the data along the line inferior to the fovea for total retinal thickness, outer retina, inner retina, RNFL, GCL, IPL, INL, OPL, ONL, ISOS layer and BM layer. The next columns show the data along the line superior to the fovea for total retinal thickness, outer retina, inner retina, RNFL, GCL, IPL, INL, OPL, ONL, ISOS layer and BM layer.
(XLSX)
